# Virtual Reality for the Assessment of Everyday Cognitive Functions in Older Adults: An Evaluation of the Virtual Reality Action Test and Two Interaction Devices in a 91-Year-Old Woman

**DOI:** 10.3389/fpsyg.2020.00123

**Published:** 2020-02-07

**Authors:** Andrea Chirico, Tania Giovannetti, Pietro Neroni, Stephanie Simone, Luigi Gallo, Federica Galli, Francesco Giancamilli, Marco Predazzi, Fabio Lucidi, Giuseppe De Pietro, Antonio Giordano

**Affiliations:** ^1^Department of Psychology of Developmental and Socialization Processes, Sapienza University of Rome, Rome, Italy; ^2^Psychology Department, Temple University, Philadelphia, PA, United States; ^3^Institute for High Performance Computing and Networking, National Research Council, Naples, Italy; ^4^Department of Engineering, Parthenope University of Naples, Naples, Italy; ^5^Fondazione Il Melo Onlus, Gallarate, Italy; ^6^Sbarro Institute for Cancer Research and Molecular Medicine, Center for Biotechnology, College of Science and Technology, Temple University, Philadelphia, PA, United States; ^7^Department of Medical Biotechnologies, University of Siena, Siena, Italy

**Keywords:** activities of daily living, everyday action, virtual reality, cognitive aging, psychometric assessment

## Abstract

Performance-based functional tests for the evaluation of daily living activities demonstrate strong psychometric properties and solve many of the limitations associated with self- and informant-report questionnaires. Virtual reality (VR) technology, which has gained interest as an effective medium for administering interventions in the context of healthcare, has the potential to minimize the time-demands associated with the administration and scoring of performance-based assessments. To date, efforts to develop VR systems for assessment of everyday function in older adults generally have relied on non-immersive systems. The aim of the present study was to evaluate the feasibility of an immersive VR environment for the assessment of everyday function in older adults. We present a detailed case report of an elderly woman who performed an everyday activity in an immersive VR context (Virtual Reality Action Test) with two different types of interaction devices (controller vs. sensor). VR performance was compared to performance of the same task with real objects outside of the VR system (Real Action Test). Comparisons were made on several dimensions, including (1) quality of task performance (e.g., order of task steps, errors, use and speed of hand movements); (2) subjective impression (e.g., attitudes), and (3) physiological markers of stress. Subjective impressions of performance with the different controllers also were compared for presence, cybersickness, and usability. Results showed that the participant was capable of using controllers and sensors to manipulate objects in a purposeful and goal-directed manner in the immersive VR paradigm. She performed the everyday task similarly across all conditions. She reported no cybersickness and even indicated that interactions in the VR environment were pleasant and relaxing. Thus, immersive VR is a feasible approach for function assessment even with older adults who might have very limited computer experience, no prior VR exposure, average educational experiences, and mild cognitive difficulties. Because of inherent limitations of single case reports (e.g., unknown generalizability, potential practice effects, etc.), group studies are needed to establish the full psychometric properties of the Virtual Reality Action Test.

## Introduction

Performance-based tests, that evaluate the ability to perform everyday tasks in the laboratory/clinic, solve many of the limitations associated with the use of self- and informant-report questionnaires of everyday functioning in people with cognitive impairment (see Giovannetti et al., 2013 for a review). Performance-based, functional tests are objective, standardized, allow a systematic comparison between individuals and provide detailed information on behaviors during the natural performance of activities. The validity of performance-based measures is supported by studies showing expected differences between clinical groups and controls (Giovannetti et al., 2002, 2008a, 2018; Schwartz et al., 2002; Allain et al., 2014; Gold et al., 2015; Rycroft et al., 2018), significant (though modest) relations with cognitive tests (Giovannetti et al., 2002, 2008a, 2018; Schwartz et al., 2002; Kessler et al., 2007; Allain et al., 2014; Rycroft et al., 2018), and informant and clinician reports of functioning (Giovannetti et al., 2002, 2008b; Schwartz et al., 2002; Allain et al., 2014). Detailed analyses of errors and error-types afforded by performance-based tests of everyday function also have promoted theoretical frameworks to better characterize the breakdown of everyday function due to cognitive impairment (see Schwartz, 2006; Giovannetti et al., 2013; for a review). Despite their validity, objectivity and potential for characterization of functional difficulties, performance-based measures have not been widely adopted in clinics or research studies, because generally they require an extraordinary effort to administer and score, especially when used to assess minor difficulties.

Virtual reality (VR) technology has recently gained interest as an effective medium for administering different interventions in the context of healthcare (Cipresso and Serino, 2014; Chirico et al., 2016; Indovina et al., 2018). Several observational studies and a small number of controlled studies have found VR to be effective for a variety of health issues (Cipresso et al., 2016). VR also has been proposed to improve clinical assessments, as automated VR systems could dramatically reduce the time required for administration and scoring traditional performance-based assessments without sacrificing ecological validity. To date, efforts to develop VR systems for assessment of function in older adults have mostly relied on non-immersive systems (Cipresso et al., 2014). In 2014, Allain et al. (2014) reported results from the Virtual Kitchen (VK), a non-immersive activity that required participants to use a mouse to select and move target objects and avoid distractor objects on a computer screen to prepare a cup of coffee. In 2019 Giovannetti et al. (2019) reported preliminary data from a modified VK, called the Virtual Kitchen Challenge (VKC), which included complex tasks to enable assessment of participants with mild cognitive difficulties and requires participants to use a touch screen interface instead of a mouse. Automated scores from the VKC were significantly associated with scores from the same tasks performed with real objects in a real kitchen.

Immersive VR systems also have been proposed to assess everyday function, as they have the advantage of creating a sense of realism or “presence” in the user. Presence is a multidimensional construct that describes the extent to which users believe and feel that they exist in the environment simulated by VR (e.g., kitchen; Diemer et al., 2015) rather than in their true physical location (e.g., clinic/lab; Witmer and Singer, 1998). Presence may be influenced by the quality of the visual scene, method of interaction/interface with the virtual environment, and other factors. Immersive VR assessments of everyday function that elicit a high degree of presence in the user might demonstrate greater ecological and predictive validity of everyday function than non-immersive tasks (Shahrbanian et al., 2012; Parsons, 2015). Although immersive systems afford greater “presence,” they also introduce unique challenges. One challenge, which is particularly salient for older adults, is managing the interface between the user and the surrounding virtual environment, because the immersive context increases the complexity of the task. Using a head-mounted display (HMD), Nolin et al. (2013) and Banville et al. (2017, 2018) implemented an immersive VR task that required participants to use the computer keyboard and mouse to sort everyday objects – a task that would be quite easy for older adults in real-life. Results showed that that older participants took more time to navigate within the virtual environment and to complete the sorting task. Also, older participants were more variable in the time required to accomplish the sorting task as compared to younger participants. These findings underscore the importance of the comfort and ease of the interface, which should feel familiar to the user and optimize mobility. Many immersive VR hardware solutions have been introduced, such as data gloves or controllers, some with haptic feedback; however, they generally prove to be too expensive and require substantial set up time. New, low-cost and ready-to-use devices, such as advanced controllers, could keep costs and administration time low and promote presence in the user during the interaction (Caggianese et al., 2019).

Advanced controllers (hereafter controllers) include buttons and tactile surfaces that are manipulated by the participant. Controllers offer indirect tracking of the position and orientation of the participant’s body. In contrast, egocentric sensors (hereafter sensors) are head-mounted small sensing devices used to detect and track the users’ hands from images acquired from the users’ point of view, directly transforming hands and finger movements into interactions with virtual objects. Both controllers and sensors allow the user to see the movement of her/his hands while being immersed in a virtual environment. A recent study comparing the most frequently used controllers (HTC Vive Controllers) and sensors (Leap Motion) with three simple manipulation tasks (i.e., select, position and rotate virtual objects) in eight participants aged 30–40 years showed an advantage for Vive Controllers, which were more stable, accurate, and easier to learn than the Leap Motion sensor (Caggianese et al., 2019).

The aim of the present study was to evaluate the feasibility of a fully immersive VR environment for the assessment of everyday function in older adults. We present a detailed case report of an elderly woman (Tina) who was selected because she represents a typical older adult with no particular computer or technological expertise and an average level of education. Tina was observed while performing an everyday activity in an immersive VR context with two different types of interfaces (controller vs. sensor). VR performance was compared against performance of the same task with real objects outside of the VR system. Comparisons were made on several dimensions, including (1) quality of task performance (e.g., order of task steps, errors, use and speed of hand movements); (2) subjective impression (e.g., attitudes, presence, cybersickness, and usability), and (3) physiological markers of stress.

## Materials and Methods

### The Participant

Tina is a 91-year-old, single women living independently in Northern Italy in a community-residence for older adults. Tina was born in Italy and is a native Italian speaker. At the time of the study she reported that she was functioning independently and had no current or past neurological or psychiatric disorders or other major medical illness (e.g., dementia, brain injury, schizophrenia, depression, etc.). She demonstrated no sensory or motor deficits that precluded interaction with a Head Mounted Display and controllers/sensors. Tina was recruited as a volunteer through an announcement made at her residence.

### Procedure

The study was approved by the Ethical Committee in the Department of Psychology of Developmental and Socialization Processes at “Sapienza” University of Rome. All procedures were completed in a single 2- to 3-h session that included the following (in order of administration): (1) informed consent obtained by the participant, (2) screening interview, (3) training on the Virtual Reality Action Test (VRAT) with controllers, (4) testing on the VRAT with controllers followed by presence and attitudes questionnaires, (5) testing on the Real Action Test followed by presence and attitudes questionnaires (6) VRAT sensor training; (7) VRAT sensor testing followed by presence and attitudes questionnaire, and (8) cognitive tests and questionnaires of mood, anxiety and everyday function.

### Performance-Based Functional Tests

The breakfast task was administered across all platforms: Real Action Test and Virtual Reality Action Test (with two different controllers). The breakfast task was selected because it has been widely studied as part of the Naturalistic Action Test (NAT), a performance-based test developed to evaluate the cognitive difficulties associated with the completion of everyday activities in people with neurologic impairment (Schwartz et al., 2002). The breakfast task requires participants to prepare a slice of toast with butter and jelly and a cup of coffee with milk and sugar while seated at a table containing a toaster, two knives, one spoon, butter in butter dish, sugar in a bowl, bottle of milk, mug filled with warm water, bread, instant coffee, jelly jar, and a napkin at the central workspace. The shape of the table and the spatial arrangement of objects was informed by procedures in the NAT manual^1^.

The breakfast task was administered in real and two different VR conditions (described below). In each condition, Tina was instructed to complete the task in silence, as quickly as possible, and without making errors. She was asked to make her movements as clear as possible and to tell the examiner when she was finished. Performance was recorded for scoring. Physiological and kinematic data were obtained while the participant completed the breakfast task according to the procedures described below.

#### Real Action Test (RAT)

**FIGURE 1:**
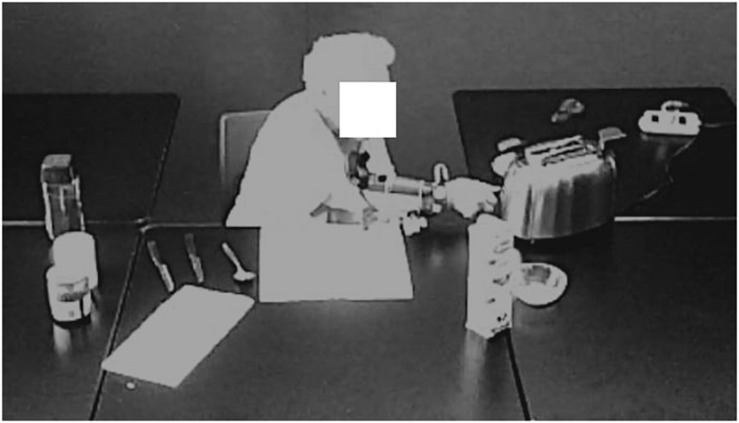
The subject (Tina) performing the Real Action Test (RAT).

The RAT required the participant to complete the breakfast task without feedback using real objects. The participant performed the RAT while wearing a smart band and wireless controllers (described below) attached to her arms to acquire kinematic and physiological data (see Figure 1).

#### Virtual Reality Action Test (VRAT)

The VRAT is a VR version of the breakfast task designed to maximize ecological validity by simulating a real kitchen and household objects. In this respect, the VRAT environment is characterized by a high degree of realism, including accurate 3D models and spatial audio. The VRAT includes automatic, real-time collection of movement data, as well as physiological and kinematic parameters (described below).

### VRAT Apparatus and Controller Conditions

The VRAT system runs on a MSI Trident Gaming Desktop, with 8GB RAM and a GTX 1060 graphic card. The HTC Vive head mounted display^2^ provides users with a fully immersive virtual environment. The HTC Vive visual system is based on two OLED displays for a total resolution of 2160 × 1200 pixels with a 110-degree FoV and a frequency of 90 Hz. The VR software was developed with Unity3D^3^, a game development platform which provides native VR support.

Interaction in the VRAT was enabled through two different input devices: (1) *controllers* – the participant used HTC Vive controllers that provided tactile feedback through vibration; (2) *sensors* – a wearable egocentric sensor, the Leap Motion Controller^4^, enabled interaction through movements of the participant’s own hands. Performance with the two different devices were tested in different conditions.

Controllers: were worn during performance of the RAT and the VRAT-controller conditions. During the RAT, participants did not interact with the controller; it was used only to collet kinematic data. However, in the VRAT, the controller was used to interact with the VR environment while the participant was in a seated position using interaction metaphors similar to those used in real-life. To make the interaction as familiar and natural as possible, we implemented the Virtual Hand metaphor (Ruddle, 2005), in which the user’s hand motions are directly mapped to the virtual hand movements. When the virtual hand reaches an object, the object is highlighted to inform the user through visual feedback that it is selected and interactable. To interact with a virtual object in the VRAT, the user is instructed to press the trigger button once the object is highlighted/selected. To end the interaction, the user is instructed to release the trigger. One advantage of the controller is that the participant is able to be tracked even when the user’s hands are not visible within the user field of view, allowing a wider measurement area. Controllers also provide users with tactile feedback through vibrations of varying intensity. However, interactions with virtual objects occur through a tool that the user must always hold in the hands, even when they are not interacting with any object, reducing the naturalness of the interaction.

Virtual Reality Action Test sensors: were used during performance of the VRAT-sensor condition. In this condition, the participant interacted with virtual objects using Leap sensors by performing a pinch gesture (i.e., moving thumb and index fingers closer until they come into contact). To release the virtual object(s) the pinch gesture is relaxed. The Leap sensor allows the user to interact with virtual objects with their own hands, without having to wear gloves or hold controllers. Unlike the controllers, the sensor is able to track the main joints of the user’s hand and replicate them in the virtual environment, increasing the hand representation and the sense of presence. However, the interaction area is limited to the tracking area of the sensor and the user’s field of view. The sensor is mounted in front of the headset; therefore, the user must keep their hands in their field of view to interact with virtual objects. Furthermore, tracking may fail if the hand is occluded by the user’s other hand or an obstacle/object in the real world.

Participants completed the RAT and both VRAT conditions while wearing a smart bracelet (Microsoft band 2) that was designed to obtain physiological measures of stress (described below).

### Software Architecture

The system was designed as a multiplayer platform: one player is the participant, who performs the task within the virtual environment, and the other player is the examiner, who configures the test, and monitors, in real time, the scores and physiological parameters of the participant. The system includes a VR module that maps the data acquired by the HMD and input devices into the corresponding virtual actions within the virtual kitchen. The game logic of the breakfast task, including the physical features and behavior of each virtual element on the table, is coded in the VR module. An error checking module has been developed for automatically detecting an error by the participant. For each participant action during the task, the error checking module considers the virtual environment state, and through a specified set of rules, interprets the participant action as either an error or correct action. Each time the participant commits an error, it notifies the logger module. The logger module acquires data from various sources (error checking module, HMD, input devices) and synchronizes them under a single time value, making it possible to link all of the separate data streams (i.e., knowing the physiological state of the participant when she/he commits an error). All information is saved as. csv files at the end of the test. The examiner interface allows the examiner to manage the test from the control panel and view errors committed by the participant as well as physiological values in real time.

### VR Training

Before each VR condition, the participant completed a brief training session with the system. Training included four mini-tasks that comprised elements of the breakfast task: (1) toast a slice of bread; (2) spread the jelly on toast; (3) add instant coffee to cup; (4) add milk to cup. The examiner controlled the presentation of each mini-task from a monitoring position.

### Quality of Task Performance

Although the VRAT includes the error monitoring module, performance quality and accuracy on the RAT and two VRAT conditions were evaluated by trained coders who viewed recordings of the participant’s performances. The following error scores were collected for each of the three conditions (RAT, VRAT-controller, VRAT-sensor):

Total overt errors – incorrect actions (commission), the failure to complete a step (omission), and off-task actions (additions) were recorded and assigned a code according to the error taxonomy shown in Table 1 (Schwartz et al., 2002).

**TABLE 1:** Error Taxonomy Used Code Performance on the RAT and VRAT conditions.

Error type		Definition	Examples
Omission		Number of steps that are not performed	Does not add coffee grounds to coffee; does not add stamp to envelope
Commission	Substitution	Similar, alternate object is used in place of target object	Spreads butter on toast with spoon instead of knife
	Sequence	Anticipation of a step; steps or subtasks performed in reverse order	Butter on bread without toasting; applies jelly on bread then applies butter
	Perseveration	A step is performed more than once or for an excessive amount of time	Adds butter/jelly repeatedly to toast
Action-Additions		Performance of an action not readily interpreted as a task step	Puts toast in creamer
Micro-errors		Initiating and terminating an incorrect action before the error is completed by reaching for, touching or picking up an object	Reaches toward, touches or moves salt but never uses the salt in during the task
Clumsy		Correct step is performed but with difficulty due to motor imprecision	Coffee jar slips out of hand

Total micro-errors – subtle, inefficient but not overtly incorrect actions; this category of errors was added to the overt error taxonomy to improve detection of subtle, inefficient behaviors in healthy people and those with mild cognitive difficulties.

Clumsy-motor imprecision errors during the execution of an accurate task step.

Code sheets with an exhaustive list of overt/micro-errors were used to promote inter-rater reliability and are included in Supplementary Material.

In addition to errors, human coders evaluated video recordings for accomplishment, time to completion and the order of task steps as follows:

Accomplishment score – an accomplishment point was assigned for each task step of the breakfast task completed without error (range = 0–16).

Overall performance score – this score combines accomplishment score with the sum of a subset of key, overt errors (Schwartz et al., 2002).

Completion Time – was recorded in seconds; timing began when the first step was initiated and ended when the participant indicated that she was finished with the task.

Order of Task Steps – In addition to coding errors and completion time, the order in which the participant completed each task step was recorded to examine similarities/differences across the RAT and VRAT conditions.

Kinematic measures were obtained by the input devices used in the RAT and VRAT conditions. During the RAT and VRAT-controller conditions, the participant wore wireless controllers, and during the VRAT-sensor condition, the participants movements were recorded by Leap Motion. Kinematic data was obtained to measure the precise movements of both the right and left hands, with an accuracy in millimeters (100 Hz). Instantaneous velocity measures greater than three meters per second were excluded to avoid noisy data due to hand tracking problems in the VRAT-sensor condition. For each condition, the following kinematic measures were obtained:

• Total hand movement, in meters.
• Average speed of the hands, in meters per second, computed as total hand movement divided by completion time.

#### Subjective Impressions

Immediately following each condition (RAT, VRAT-controller, VRAT-sensor), the participant used a five-point scale to describe her reaction to the test condition on the following five items/dimensions: useless/useful, not pleasant/pleasant, boring/funny, tiring/resting, stressing/relaxing. Item scores were aggregated into a single score, ranging from 5 to 25, for which higher values indicated more positive attitudes about the test condition. This scale was created by the authors of the study according to procedures described by Ajzen (1991); see Supplementary Material.

### Physiological Measures of Stress

To compare indicators of stress during each testing condition, physiological data were recorded via a smart bracelet (Microsoft band 2)^5^ worn by the participant while completing the RAT and both VRAT conditions. Kubios software (Tarvainen et al., 2014) was used to obtain the following variables:

Heart rate (bpm, 1 Hz),
Galvanic Skin Response (kohms, 0,2/5 Hz),
R–R interval (i.e., time between heart beats; seconds, variable frequency),
skin temperature (degrees centigrade, 0,033 Hz).

To correct for artifacts, particularly in the measure of heart rate variability (RR interval), a threshold-based algorithm was applied that compares every RR interval value against a local average interval, obtained by median filtering the RR interval time series. RR interval values that differ from the local average of a specified threshold value (i.e., 0.45 s) are marked as artifact and replaced using cubic spline interpolation.

Physiologic variables (i.e., Heart rate, Galvanic Skin Response, Skin temperature) were used to calculate an index of cardiovascular system stress, called Baevsky’s stress index (Baevsky and Berseneva, 2008). The Baevsky’s stress index is strongly linked to sympathetic nervous activity and increases during stressful situations. Physiologic data were stored on .csv files and although they may be combined with the test start time to synchronize physiological and kinematic information, for the current study, physiologic data were aggregated and averaged for each test condition to obtain an overall stress index per condition (RAT, VRAT-controller, VRAT-sensor).

### VRAT Presence, Cybersickness, and Usability

The following questionnaires were administered immediately following performance on the VRAT-controllers and VRAT-sensors conditions.

#### Presence Questionnaire (PQ)

The Italian version of PQ was administered to the participant in this study (Scheuchenpflug et al., 2003). The PQ required the participant to use a seven-point scale to rate her experience with each condition on 28 items focused on the following features: Realism (7 items); Possibility to act (4 Items); Quality of interface (3 Items); Possibility to examine (3 items); Self-evaluation of performance (2 Items) (Witmer and Singer, 1998; Slater, 2002; Witmer et al., 2005). Strong internal reliability has been reported (0.88) for the total score.

#### Cybersickness Symptoms

The Virtual Reality Symptom Questionnaire (VRSQ), developed by Ames in 2005 (Ames et al., 2005), was administered immediately after the VRAT-controllers condition and the VRAT-sensors condition to evaluate symptoms of cybersickness, a type of motion sickness caused by exposure to VR. The questionnaire assesses eight general physical side effects (general discomfort, fatigue, boredom, drowsiness, headache, dizziness, concentration difficulties, and nausea) and five visual effects (tired eyes, aching eyes, eyestrain, blurred vision, and difficulties focusing) on a seven-point scale (0–6), with 0-scores indicating no symptoms and higher scores indicating more severe symptoms. In the validation study, only symptoms that met a minimum correlation coefficient value of 0.2 with the total score were included in the final measure. The Italian version of the VRSQ (Solimini et al., 2011) was used with the participant in this study.

#### System Usability Scale (SUS)

The SUS is a 10-item measure that required the participant to use a five-point scale ranging from strongly disagree to strongly agree to indicate the extent to which they agree/disagree with positive and negative statements about the VRAT-controller and VRAT-sensor conditions (Brooke, 1996). SUS responses were transformed to a single score ranging from 0 to 100, with higher scores reflecting more favorable usability. The SUS is considered a robust measure of system usability (Bangor et al., 2008), even with a small sample size (Tullis and Stetson, 2004). The Italian version of the SUS was used in this study (Borsci et al., 2009).

### Mood, Anxiety, and Cognition

Questionnaires of mood and anxiety symptoms, disposition toward immersive tendencies, and cognitive and functional abilities as well as neuropsychological tests of global and specific cognitive abilities were administered by a trained psychologist (AC). When available, Italian validated versions of questionnaires/tests were used; other measures were translated using a back-translation procedure (see Table 2).

**TABLE 2:** Mood and neuropsychological tests performed to characterize the participant.

Variable	Test	Original scale citation	Italian scale used for the study	Validity/Reliability of the instrument
Depression	Geriatric Depression Scale (GDS)	Yesavage et al., 1982	Galeoto et al., 2018	Cronbach’s Alpha scored 0.84 in the Italian validated study (Galeoto et al., 2018)
Anxiety	Geriatric Anxiety Scale	Segal et al., 2010	Gatti et al., 2018	Cronbach’s Alpha of the Italian scale was = 0.88 (Gatti et al., 2018)
Cognitive abilities	The Everyday Cognition scale short form 12 (ECOG SF12)	Farias et al., 2011	Back Translation procedure has been made for the study purposes	E-Cog has been reported to have high internal consistency (α = 0.96). Additionally, the scale demonstrates good test–retest reliability (*r* = 0.82) (Farias et al., 2011)
Functional activities	Functional Activity Questionnaire (FAQ)	Pfeffer et al., 1982	Stancati and Salussi, 2001	The scale has been reported to have high internal consistency (α > 0.90) (Pfeffer et al., 1982)
Daily living activities	The Activities of Daily Living-Prevention Instrument (ADL-PI)	Galasko et al., 2006	Back Translation procedure has been made for the study purposes	Test–Retest reliability: was *r* = 0.74 (Galasko et al., 2006)
Education	Brief Intelligence Test (Test di Intelligenza Breve; TIB)	Colombo et al., 2002	Original scale is in Italian	Cronbach’s Alpha scored 0.91 (Colombo et al., 2002)
Visual memory	Brief Visual Memory Test Revised (BVMT – R)	Benedict et al., 1996	Argento et al., 2016	Test–retest reliability coefficients ranged from 0.60 for Trial 1 to 0.84 for Trial 3 (Argento et al., 2016)
Verbal fluency	Category Fluency	Sivan and Benton, 1984	Novelli et al., 1986	Test–retest reliability coefficients for the scale was > 0.75 (Kingery et al., 2011)
Processing speed	Trail Making Test-Part B	Armitage, 1946	Gaudino et al., 1995	Validity of the test has been extensively discussed and confirmed (for an extensive review see Sánchez-Cubillo et al., 2009)
Working memory	Digit Span backward	Wechsler et al., 2008	Monaco et al., 2013	The test reliability scored 0.89 (Orsini and Pezzuti, 2015)
Processing speed and visual perception	Symbol search	Wechsler et al., 2008	Orsini and Pezzuti, 2015	The test reliability scored 0.88 (Orsini and Pezzuti, 2015)
Personal disposition toward immersion	Immersive Tendencies Questionnaire	Witmer and Singer, 1998	Scheuchenpflug et al., 2003	The scale reliability scored 0.81 (Witmer and Singer, 1998)

### Analysis Plan

Descriptive analyses of questionnaires and cognitive tests were performed to characterize the participant. Cognitive test scores also were evaluated by calculating the standardized (*Z*) score for the participant relative to normative data, using samples that were comparable to the age and education level of the participant. The following formula was used to calculate the *Z*-score (participant’s raw test score – mean of the normative sample/E.S. of the normative sample).

Descriptive data from the RAT, VRAT-controllers, and VRAT-sensors were obtained to compare performance across the testing conditions on measures of (1) the quality of task performance (e.g., errors, accomplishment, time to completion, order of task steps, errors, use and speed of hand movements, etc.); (2) subjective impressions (e.g., attitudes, presence, cybersickness, and usability), and (3) physiological markers of stress.

## Results

### Characterization of the Participant

#### Mood Status

Tina’s report of depression (Geriatric Depression Scale = 4) and anxiety (Geriatric Anxiety Scale = 12) symptoms was well within the non-clinical range (cut-off scores: GDI > 11; GAI > 17) (Yesavage et al., 1982; Segal et al., 2010; Gould et al., 2014; Galeoto et al., 2018; Gatti et al., 2018).

#### Cognitive Testing

Raw cognitive test scores along with age- and education-adjusted normative-based *Z*-scores are reported in Table 3. Tina’s overall cognitive status, as measured by the MMSE was well within the range of healthy, non-demented people. Scores on most tests of specific abilities fell within the average range, including tests of verbal episodic memory, processing speed, executive functions, and verbal fluency. She performed in the high average range on a verbal test of executive function and in the low average range on a test of visual episodic memory (immediate and delayed free recall).

**TABLE 3:** Characterization of the participant adjusted for age and education.

Test	Subtest	Raw score	Standard score (*z*-score)	Qualitative descriptor
MMSE		29/30		Within normal limits
**BVMT^1^**				
	Trial 1	3	–4.66	Impaired
	Trial 2	4	–3.79	Impaired
	Trial 3	8	–1.21	Low Average
	Learning trial	5	–0.18	Average
	Delayed recall trial	5	–2.95	Impaired
**RAVLT^2^**				
	Total score	45	1.52	Average
	Delayed recall	8	0.20	Average
	Recognition hits	15	2.25	Average
Symbol search^3^		19	1.67	Average
TMT-B^4^		298.21	0.48	Average
DIGIT SPAN backward^5^		5	3.37	High average
Fluency global^6^ (Categories: car brand, animal, fruit)		52	2.52	Average
Questionnaires pertaining to cognition, everyday function, and immersive tendencies FAQ		6		
ECOG-Short Form		1.75		
ADL PI		22		
**ITQ**				
	Focus subscale	31		
	Involvement subscale	21		

*BVMT, Brief Visual Memory Test Revised; RAVLT, Rey Auditory Learning Test; TMT-B, Trail Making Test-Part B; FAQ, Functional Activity Questionnaire; ECOG. SF-12, The Everyday Cognition scale short form 12; ADL-PI, The Activities of Daily Living-Prevention Instrument; ITQ, Immersive Tendencies Questionnaire. ^1^Normative data for the Italian population (Argento et al., 2016); ^2^Normative data (Carlesimo et al., 2002); ^3^Normative data for the Italian population (Orsini and Pezzuti, 2015); ^4^Normative data for the Italian population (Giovagnoli et al., 1996); ^5^Normative data for the Italian population (Monaco et al., 2013); ^6^Normative data for the Italian population (Novelli et al., 1986).*

On questionnaires of cognitive and functional abilities, Tina reported no significant change in her cognitive abilities as compared to 10 years ago [The ECOG SF12 = 1.75, cut-off score = 2.30 (Farias et al., 2008)] and minimal functional difficulties within the normal range [FAQ (score = 6) and the ADL-PI (score = 22)].

On a questionnaire pertaining to one’s personal disposition toward immersion (ITQ), Tina reported an average level of immersion in terms of ability to focus and to become deeply involved in activities (Witmer and Singer, 1998).

### Comparisons Across the RAT, VRAT-Controllers, and VRAT-Sensors

#### Performance Quality

As shown in Table 4, Tina made few errors on the breakfast task across all conditions, with most errors on the VRAT-controllers condition. She made no clumsy errors on the RAT, but an equal number of clumsy errors on both VRAT conditions. The Overall Performance Score, which considers accomplishment and the performance of key overt errors was identical across the conditions. Time to completion, also shown in Table 4, revealed a longer completion time for the VRAT – controllers than the other two conditions.

**TABLE 4:** Quality task performance, kinematic, physiological data and system usability.

	RAT	VRAT- controllers	VRAT- sensors
**Performance analysis**			
Accomplishment (%)	100	100	100
Total overt errors	0	1	1
Total micro-errors	3	6	0
Total clumsy errors	0	7	7
Overall Performance (Max = 6)	6	6	6
Completion Time	108.72	203.74	165.81
**Kinematic analysis**			
Total hand movement (m)			
Right hand	22.91	43.97	28.77
Left hand	11.3	6.71	11.6
Total hand speed (m/s)			
Right hand	0.21	0.21	0.17
Left hand	0.1	0.03	0.07
**Physiological data – Mean (SD)**			
Baevsky’s stress index	4.1	4.9	6.2
Heart rate (bpm)	72.34 (1.54)	68.79 (3.63)	78.15 (2.29)
Galvanic skin response (kohms)	2467 (200)	4714 (820)	893 (44)
Skin temperature (celsius degree)	35.16 (0.07)	34.99 (0.13)	35.22 (0.02)
**System usability**			
System usability (SUS)		62.5/100	50/100
**Sense of presence**			
Sense of presence global score (PQ)		113/126	100/126
Realism – subscale		39/49	37/49
Possibility to act – PQ subscale		27/28	18/28
Quality of the interface – PQ subscale		16/21	18/21
Possibility to examine – PQ subscale		19/21	17/21
Self-Evaluation of the performance – PQ subscale		12/14	10/14

*SUS, System Usability Scale; PQ, Presence Questionnaire.*

A qualitative analysis of the order in which steps were performed showed remarkable consistency. Task steps were performed in the following order across all three conditions: take bread, place bread in toaster, turn on toaster, wait for bread to toast, remove bread from toaster, add butter to toast, add jelly to toast, add coffee to mug, add milk to mug, add sugar to mug. The final step of stirring the coffee was completed only in the RAT. Tina did not stir the virtual coffee mug in either the VRAT-controller or VRAT-sensor condition; this was coded as an overt (omission) error in both of the VRAT conditions.

### Kinematic Results

Hand movements and average hand speed are also shown in Table 4. The same pattern of hand movement distance and speed was observed across all conditions – the right hand made more and faster movements than the left hand. There were few differences across conditions, except for a greater reliance on the right hand in the VRAT-controller condition.

**FIGURE 2:**
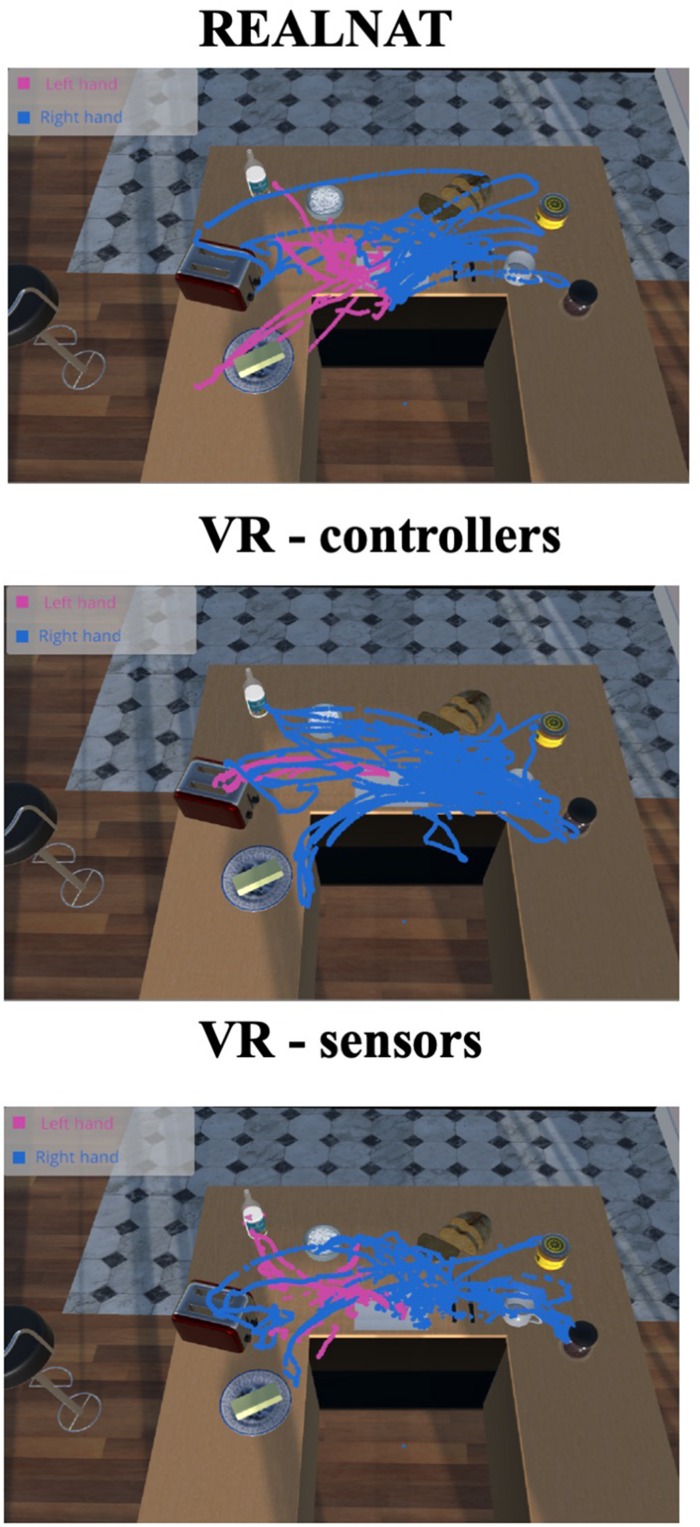
Hands heat map for the three different experimental conditions.

A heatmap showing the paths of the right and left hand during each condition is shown in Figure 2. Note that the heatmap for the RAT was superimposed on a virtual display for presentation purposes only. The participant actually completed the RAT using real objects as shown in Figure 1. The heat maps illustrate subtle differences across conditions. In the RAT, the participant used both hands to perform the steps (i.e., using her left hand to grab the milk bottle, the butter dish and sugar bowl), with each hand performing tasks in the corresponding hemispace. In the VRAT conditions, particularly in the VRAT-controller condition, the participant used the dominant, right hand more frequently, even when completing subtasks in the opposite (left) hemispace.

### Physiological Markers

As expected, the lowest stress index was obtained during the RAT (stress index = 4.1); followed by the VRAT-controller (stress index = 4.9) and VRAT-sensor (stress index = 6.2). This result suggests that the participant felt more comfortable with controllers rather than in the sensor condition without the controllers (Table 4).

### Subjective Impressions

As shown in Table 4, Tina reported the most positive attitude toward the VRAT-controllers (Total = 25/25) and the RAT (Total = 24/25). She indicated the lowest score for the VRAT-sensor condition (16/25), as she reported that the VRAT-sensor condition was less “pleasant,” “funny,” “resting,” and “relaxing” (each scored 3 out of 5).

Measures of presence, cybersickness, and usability were obtained after each of the VRAT conditions. As shown in Table 4, Tina reported a stronger feeling of presence in the VRAT-controllers than in the VRAT-sensors condition (PQ). Scores for each of the PQ subscales, except the “quality of interface” scale were all higher in the VRAT-controller condition (see Table 4). Tina reported no symptoms of cybersickness on VRSQ for either condition (Ames et al., 2005). Finally, Tina reported higher usability ratings for the VRAT-controllers than the VRAT-sensors condition.

## Discussion

This paper reports the detailed analysis of a 91-year old woman’s (Tina) performance of a real (RAT) and immersive VR breakfast task (VRAT) to evaluate the feasibility of immersive VR for the assessment of everyday function in older adults. Two different VR interfaces were examined: controllers and sensors. Results showed similarities in performance quality, stress, and subjective reports between the RAT and both VRAT conditions, as well as positive ratings and no cybersickness for either VR condition. Taken together the results demonstrate the feasibility of immersive VR for function assessment in older adults and suggest the potential of the validity of this method.

Our results clearly demonstrate the feasibility of immersive VR for function assessment, even in an older adult with very limited computer experience, no prior VR exposure, average educational experiences, and mild cognitive difficulties. The participant was capable of using controllers and sensors to manipulate objects in a purposeful and goal-directed manner in the VR paradigm. She reported no cybersickness and even indicated that interactions in the VR environment were pleasant and relaxing.

Our results also suggest the potential validity of the VR paradigm, as overall performance and accomplishment scores were similar, and task steps were completed in exactly the same order across conditions, even though there were numerous opportunities for variation in the order of steps (e.g., coffee could have been made before toast and the order of cream and sugar and butter and jelly was not fixed). Kinematic analyses also were generally comparable between the real (RAT) and the VRAT-sensor condition, and the participant reported positive attitudes toward real (RAT) and both VRAT tasks. These similarities are striking considering that immersive VR was completely unfamiliar to the participant.

Some important differences between the real and VR paradigms were observed and should inform future research. For example, the participant required less time and demonstrated a lower stress index while completing the real breakfast task (RAT). She also demonstrated fewer clumsy errors in the real task as compared to both VRAT conditions. These differences suggest that the real condition was considerably easier for the participant. Training with the VR controllers and sensors was minimal in the present study, and the participant had no prior experience with VR. Future studies that use VR with older adults should consider including more training to determine whether increased familiarity with the VR environment and practice with VR controllers/sensors may further reduce differences between real and virtual everyday task performance.

In contrast to past research with healthy participants showing advantages with controllers (Caggianese et al., 2019), our results do not clearly indicate which VR interface is best for the assessment of function in older adults, as each interface showed different strengths and weaknesses. When using the controllers, the participant made more micro-errors, and kinematic analyses showed a pattern of hand use that was dissimilar from performance on the real task, such that she appeared to favor her dominant (right) hand for completing the tasks in the VRAT-controller condition. However, she subjectively reported that she preferred the controllers, with higher ratings for usability and positive attitude toward the VRAT-controllers condition. Physiological indicators also reflected lower stress when she used the controllers (VRAT-controllers) than when she used the sensors (VRAT-sensors). By contrast, with the sensors, the participant showed a more natural pattern of use of the right and left hands (see kinematic data). Taken together, the results suggest that if problems in precisely controlling movements in the sensor interface could be addressed in future research, the sensor interface has potential to offer more accurate and naturalistic assessments of everyday function for older adults than controllers.

There are several limitations to acknowledge. First, the extent to which the results are influenced by order effects cannot be determined from this single case report. Future studies should control for and examine task order and practice effects on virtual and real everyday tasks. Future studies with more participants are needed to determine whether our results are generalizable and to establish the full psychometric properties of the VRAT.

In conclusion, our results support the feasibility of immersive VR as a tool to evaluate everyday function in older adults considering also the evaluated safety of the technology as suggested by a recent meta-analysis (Kourtesis et al., 2019). The results also provide guidance on considerations for VR interfaces (sensors vs. controllers). Because of its strong potential to offer objective, sensitive and standardized assessment of everyday function in older adults and a wide range of clinical populations future research on VR assessments is needed to identify optimal interfaces and procedures, compare the utility against non-immersive VR methods (Allain et al., 2014; Giovannetti et al., 2018), and ultimately establish the psychometric properties of immersive VR measures of everyday function. Moreover, the potential for immersive VR systems to offer interventions that might improve everyday functioning and promote independence should be explored (Banville et al., 2018; Foloppe et al., 2018).

## Data Availability Statement

The raw data supporting the conclusions of this article will be made available by the authors, without undue reservation, to any qualified researcher.

## Ethics Statement

The studies involving human participants were reviewed and approved by the Ethical Committee – Department of Psychology of Developmental and Socialization Processes, Sapienza University of Rome, Rome, Italy. The patients/participants provided their written informed consent to participate in this study. Written informed consent was obtained from the individual(s) for the publication of any potentially identifiable images or data included in the manuscript.

## Author Contributions

All authors conceived and planned the experiments, contributed to the interpretation of the results, provided critical feedback, and helped to shape the research, analysis, and manuscript. AC, FeG, FrG, and PN carried out the experiments. PN and LG programmed the software. AC managed and analyzed data. TG and SS coded visual data. AG and TG took the lead in writing the manuscript.

## Conflict of Interest

The authors declare that the research was conducted in the absence of any commercial or financial relationships that could be construed as a potential conflict of interest.

## Footnotes

1 https://mrri.org/wp-content/uploads/2016/01/NATManual.pdf

2 https://www.vive.com/eu/

3 https://unity.com/

4 https://www.leapmotion.com/

5 https://support.microsoft.com/it-it/help/4000323/band-hardware-sensors
