# Supplementary material for: Virtual Reality for the Assessment of Everyday Cognitive Functions in Older Adults: An Evaluation of the Virtual Reality Action Test and Two Interaction Devices in a 91-Year-Old Woman
Source: Front Psychol. 2020 Feb 7;11:123. doi: 10.3389/fpsyg.2020.00123 (PMC7029745; doi:10.3389/fpsyg.2020.00123)
Supplement: Supplementary file 1 [file Table_1.DOCX]

**SUPPLEMENTARY MATERIAL**

**List of errors.**

**Omission**

Omit of accomplish the following steps:

1. Take bread and place into toaster
2. Turn on toaster
3. Wait 10 seconds
4. Remove toast from toaster and place on the towel
5. Scoop butter
6. Spread butter on toast
7. Open jelly
8. Scoop jelly
9. Spread jelly on toast
10. Open instant coffee
11. Scoop instant coffee
12. Add instant coffee to mug
13. Scoop sugar
14. Add sugar to mug
15. Add milk to mug

**Anticipation**

1. Toaster on without bread in
2. Butter on without toast bread
3. Jelly on without toast bread
4. Jelly on without butter on

**Perseveration**

1. Toaster more than 1 slice of bread
2. Operate toaster repeatedly
3. Apply butter perseveratively
4. Apply jelly perseveratively
5. Apply sugar perseveratively
6. Apply coffee perseveratively

**Reversal**

1. Toaster on, then bread in
2. Bread out then toaster on
3. Apply butter then toast bread
4. Apply jelly then toast bread
5. Apply jelly, then butter

**Substitution**

1. Spread butter with spoon
2. Spread jelly with spoon
3. Add sugar with knife
4. Add coffee with knife
5. Apply butter into mug
6. Apply jelly into mug
7. Apply sugar on toast
8. Apply coffee on toast
9. Apply milk on toast
10. Apply coffee on sugar bowl
11. Apply milk on sugar bowl
12. Add sugar wuth wood spoon

**ATTITUDES TOWARD TESTING CONDITION**

**(scale used after each of the three conditions)**

I believe that performing this test would be:

| Unpleasant | 1 | 2 | 3 | 4 | 5 | Pleasant |
| --- | --- | --- | --- | --- | --- | --- |
| Boring | 1 | 2 | 3 | 4 | 5 | Funny |
| Useless | 1 | 2 | 3 | 4 | 5 | Useful |
| Tiring | 1 | 2 | 3 | 4 | 5 | Resting |
| Stressing | 1 | 2 | 3 | 4 | 5 | Relaxing |

**PHISYOLOGICAL DATA**

Below are the graphs of the physiological data collected during the breakfast task. Inside the graphs there are labels, which indicate the activity that the participant was performing in that specific time interval.

**Heart rate in NATREAL test**

**
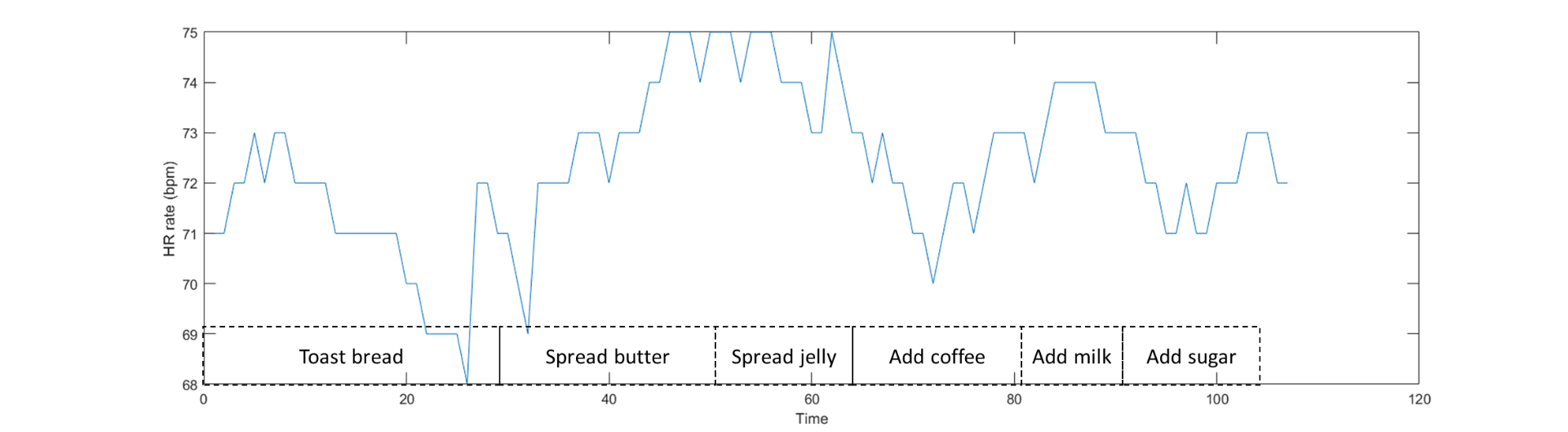
**

**Heart rate in VR test**

**
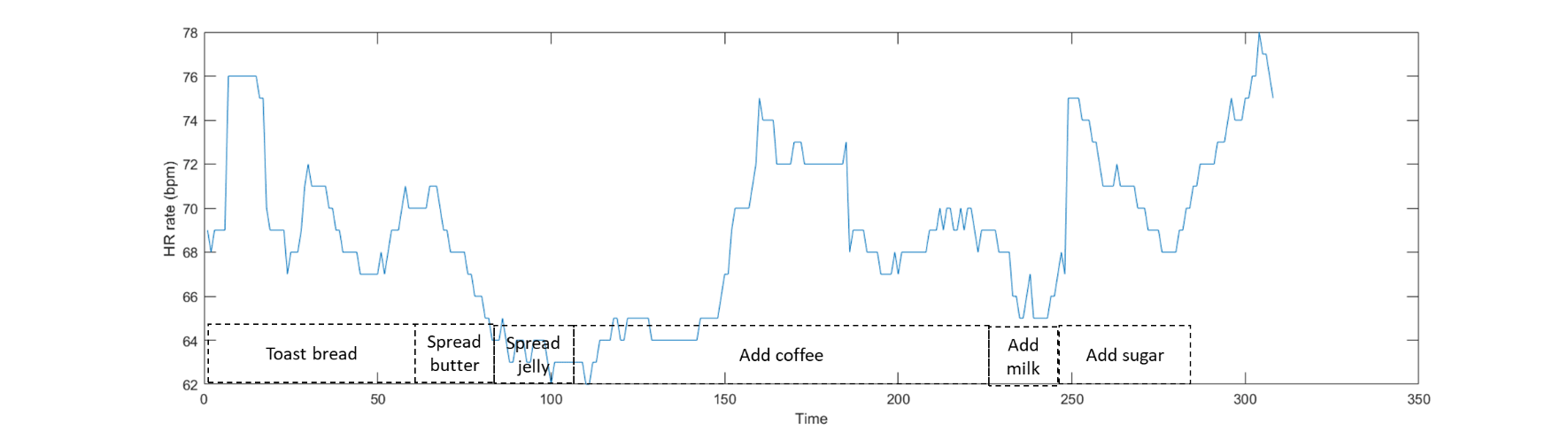
**

**Heart rate in LEAP test**

**
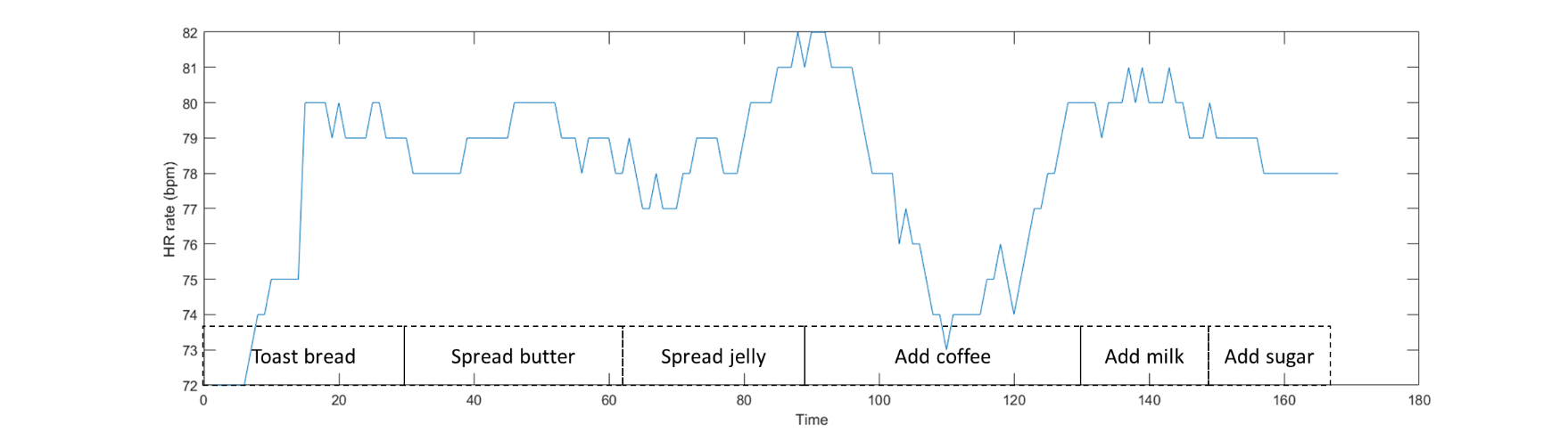
**

**Galvanic skin response in NATREAL test**

**
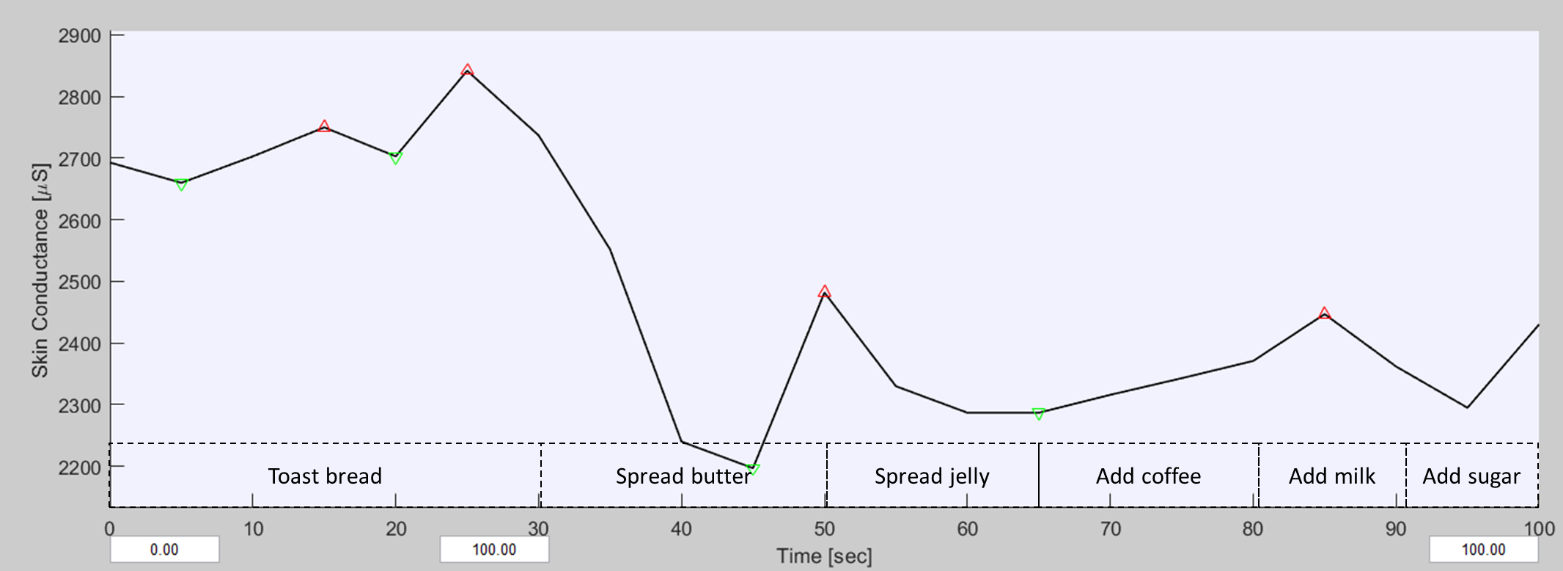
**

**Galvanic skin response in VR test**

**
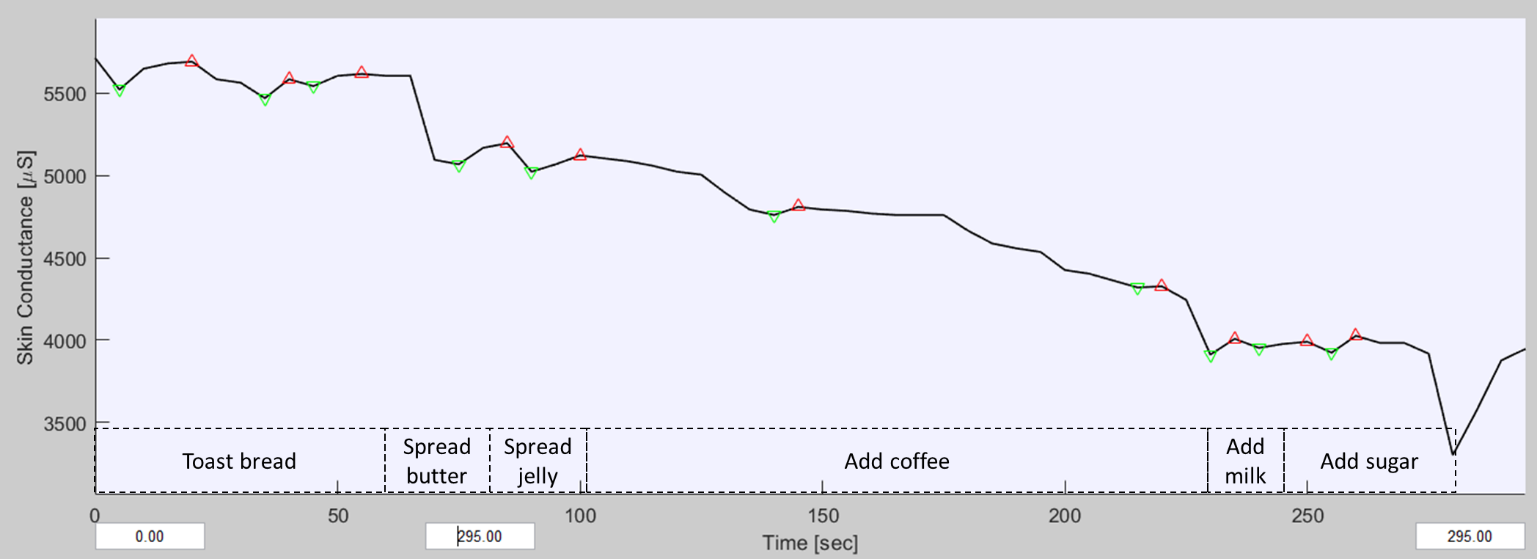
**

**Galvanic skin response in LEAP test**

**
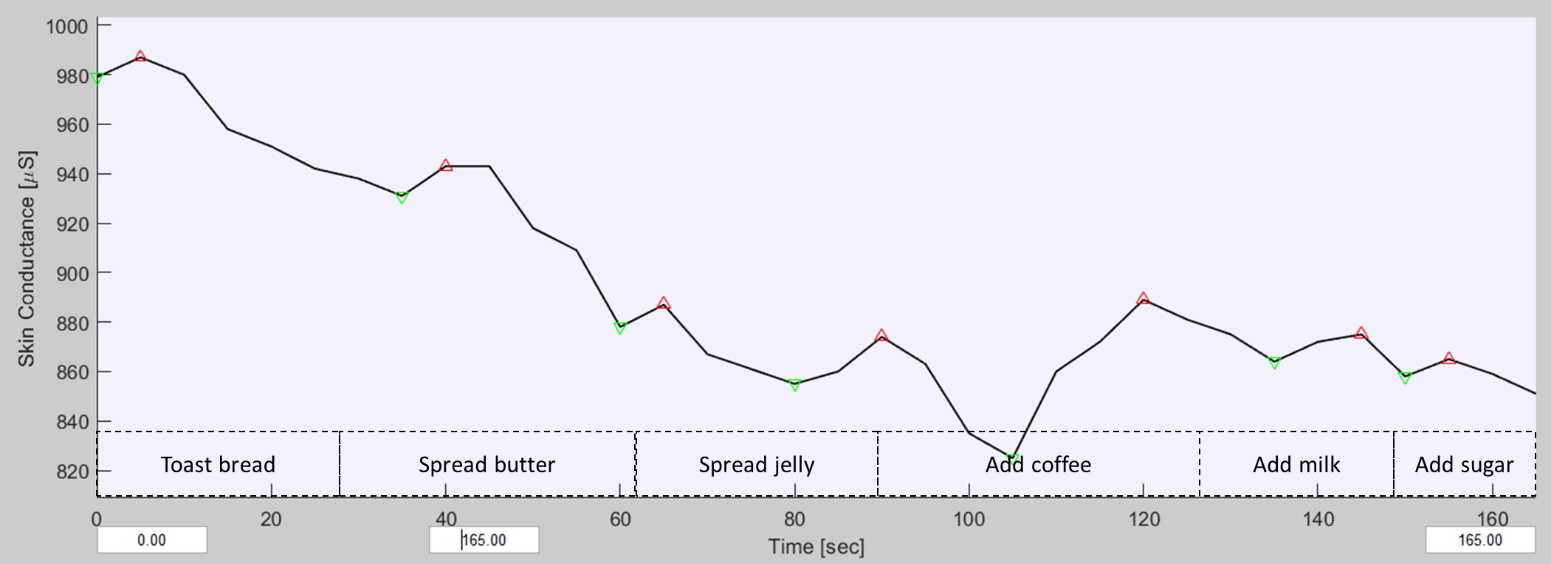
**
